# Seasonal Variation in Wild *Rosmarinus officinalis* L.: Phytochemicals and Their Multifunctional Potential Against Metabolic Disorders

**DOI:** 10.3390/molecules31020220

**Published:** 2026-01-08

**Authors:** Khaled Kherraz, Khalil Guelifet, Mokhtar Benmohamed, Luca Rastrelli, Latifa Khattabi, Afaf Khadra Bendrihem, Abderrazek Ferhat, Mohamed Amine Ferhat, Khaled Aggoun, Duygu Aygünes Jafari, Barbara Sawicka, Lilya Harchaoui, Wafa Zahnit, Azzeddine Zeraib, Mohammed Messaoudi

**Affiliations:** 1 Laboratory of Ethnobotany and Natural Substances, Higher Normal School, Vieux-Kouba, Algiers 16050, Algeria; 2 Laboratory of Research on Bioactive Products and Biomass Valorization, Department of Chemistry, Higher Normal School of Kouba (ENS), P.O. Box 92, Vieux-Kouba, Algiers 16308, Algeria; khalil.guelifet@g.ens-kouba.dz (K.G.); ferhatamine100@yahoo.fr (M.A.F.); 3 Laboratory of Fundamental Sciences, University Amar Télidji of Laghouat, Road of Ghardaïa, P.O. Box 37G, Laghouat 03000, Algeria; msbm1447@gmail.com; 4 Department of Pharmacy, University of Salerno, Via Giovanni Paolo II, 132, Fisciano, 84084 Salerno, Italy; 5 National Biodiversity Future Center (NBFC), 90133 Palermo, Italy; 6 Biotechnology Research Center (CRBt), Constantine 25000, Algeria; l.khattabi@crbt.dz; 7 Biotechnology, Water, Environment and Health Laboratory, Faculty of Natural and Life Sciences, University of Abbes Laghrour, Khenchela 40000, Algeria; afaf.bendrihem5@gmail.com (A.K.B.); azzeddinezeraib@gmail.com (A.Z.); 8 Laboratory of Research in Science and Environment, Bioresources, Geochemistry-Physics, Legislation and Socio-Economic Development, Faculty of Sciences and Technology, University of Tamanghasset, Sersouf, Tamanrasset 10034, Algeria; ferhat_abdou@yahoo.fr; 9 Laboratory of Organic Materials and Heterochemistry, Echahid Cheikh Larbi Tebessi University, Constantine Road, Tebessa 12002, Algeria; khaled.aggoun@univ-tebessa.dz; 10 Faculty of Medicine, Department of Medical Biology, Ege University, 35100 Izmir, Turkey; duygujafari@gmail.com; 11 Department of Plant Production Technology and Commoditties Science, University of Life Sciences in Lublin, Akademicka 15 Str., 20-950 Lublin, Poland; barbara.sawicka@gmail.com; 12 Faculty of Biological Sciences, University of Science and Technology Houari Boumediene, Algiers 16111, Algeria; lilya.h@live.fr; 13 Department of Chemistry, Faculty of Sciences, University of Ferhat ABBAS Setif 1, El Bez 19000, Algeria; zahnit_07_hanane@outlook.fr; 14 Laboratory N—Body and Structure of Matter, Ecole Normale Supérieure, P.O. Box 92, Vieux-Kouba, Algiers 16050, Algeria

**Keywords:** *Rosmarinus officinalis*, phenolic compounds, seasonal variation, antioxidant activity, anti-inflammatory activity, α-amylase inhibition, metabolic disorders

## Abstract

This investigation explored how seasonal variation affects the phytochemical composition and biological potential of *Rosmarinus officinalis* L., a widely used aromatic and medicinal plant. Aerial parts collected during spring, summer, autumn, and winter were extracted with ethanol and analyzed using LC-ESI-MS/MS, while total phenolic (TPC) and flavonoid (TFC) contents were determined spectrophotometrically. The extracts were evaluated for antioxidant, anti-inflammatory, enzyme inhibitory, analgesic, antimicrobial, cytotoxic, and photoprotective properties. Major constituents identified in all seasons included luteolin, kaempferol, rutin, and biochanin A. The autumn extract contained the highest phenolic (353.21 ± 4.05 µg GAE/mg) and flavonoid (190.11 ± 5.65 µg QE/mg) levels. Antioxidant assays revealed that the autumn extract had the strongest DPPH radical scavenging activity (IC_50_ = 24.72 ± 0.16 µg/mL), while the spring extract exhibited the greatest reducing power (A_0.5_ = 7.62 ± 0.30 µg/mL). The winter extract demonstrated superior anti-inflammatory activity (IC_50_ = 28.60 ± 2.84 µg/mL), exceeding the reference drug diclofenac. Only the spring extract inhibited urease (IC_50_ = 62.26 ± 0.58 µg/mL) and moderately inhibited α-amylase. All seasonal extracts showed notable photoprotective potential, with SPF values between 25.18 and 32.46, well above the recommended minimum. The spring extract also presented strong analgesic activity and no acute toxicity up to 2000 mg/kg. Antimicrobial effects were weak, limited to slight inhibition of *Staphylococcus aureus*, while moderate cytotoxicity was observed against MCF-7 and MDA-MB-231 breast cancer cells. Overall, seasonal variation significantly influenced the chemical profile and bioactivities of *R. officinalis*, with autumn and spring identified as the most suitable harvesting periods for pharmaceutical and cosmetic applications.

## 1. Introduction

Medicinal plants are among the oldest natural resources utilized by humans for the treatment and prevention of various ailments. Their use has remained prevalent throughout different historical periods due to their accessibility, relative safety, and diverse health benefits [1]. These plants constitute a biological treasure trove rich in bioactive compounds that have demonstrated efficacy in managing a wide array of conditions, ranging from mild disorders to chronic and complex diseases. In recent decades [2], global interest in medicinal plants has grown significantly, both in scientific and industrial circles, driven by the growing demand for natural alternatives to synthetic drugs, which are often associated with adverse side effects. Advances in chemical and biological analysis techniques have further facilitated the identification of plant constituents and the elucidation of their biochemical mechanisms, thus promoting their integration into complementary and preventive medicine programs and enabling their utilization in pharmaceutical, food, and cosmetic industries [3].

At the core of this scientific interest are phenolic compounds, which represent one of the most important chemical groups responsible for the bioactivity of medicinal plants. These compounds belong to a class of secondary metabolites that serve as defensive agents in plants against harmful environmental factors such as oxidative stress, ultraviolet radiation, and microbial attack [4]. The hydroxylated aromatic structure of phenolic compounds underlies their capacity to regulate redox homeostasis, limit oxidative injury, and attenuate inflammatory mediators, functions that are directly relevant to metabolic syndrome and related cardiometabolic conditions [5]. Numerous studies have shown that the biological effectiveness of plants is closely related to the quality and quantity of these compounds, particularly flavonoids, phenolic acids, and condensed polyphenols [6], which have exhibited antioxidant, anticancer [7], anti-inflammatory, antibacterial, anticoagulant, and antidiabetic properties [8]. These mechanisms are particularly relevant in the context of metabolic disorders, where oxidative stress, chronic low-grade inflammation, and enzyme dysregulation play central roles in the progression of insulin resistance and associated cardiometabolic complications. Moreover, the biosynthesis and accumulation of these compounds in plants are influenced by several environmental factors, including light, temperature, soil, and season, making it essential to understand these variations to ensure the efficacy of plant extracts [9].

Among the most prominent medicinal plants that have garnered increasing scientific interest is *Rosmarinus officinalis* L. (rosemary), an aromatic evergreen shrub belonging to the Lamiaceae family. Native to the Mediterranean basin [10], *R. officinalis* has long been used in traditional medicine to treat various ailments, including stomach aches, headaches, dysmenorrhea, spasms, rheumatic pain, epilepsy, nervous agitation, mental and physical fatigue, depression, hysteria, and to enhance memory [11]. It is also widely used in culinary applications as a natural flavoring and preservative [12]. *R. officinalis* contains a wide range of active compounds, notably phenolic acids such as rosmarinic acid and carnosic acid, which collectively contribute to its broad spectrum of biological activities [13], including antioxidant [14], antimicrobial [15], and anti-inflammatory effects. Due to this combination of antioxidant, anti-inflammatory, and enzyme-modulating activities, *R. officinalis* has emerged as a promising phytochemical source for the prevention and management of metabolic syndrome and related disorders. In Algeria, *R. officinalis* is a wild plant commonly found in many regions, especially in the northern part of the country, where it grows spontaneously in forests and mountainous areas [16]. It is traditionally collected for use in folk medicine. Recent chemical and biological studies have highlighted the high value of this plant in terms of its phenolic profile and biological activity [17]. However, most of these studies have not addressed the impact of seasonal variation on its chemical composition and bioactivities, despite the crucial role of seasonality in determining optimal harvesting times and maximizing the plant’s potential [18,19].

Based on these considerations, the present study aims to evaluate the seasonal influence on the chemical and biological properties of *R. officinalis* extracts through a comprehensive analysis. This includes the identification of the chemical profile, quantification of total phenolic and flavonoid contents, and the evaluation of various health-related biological activities. These activities encompass antioxidant capacity assessed using multiple assays (DPPH, ABTS, FRAP, ADS, and SNP), anti-inflammatory potential evaluated through both in vitro and in vivo models, inhibition of α-amylase and urease enzymes associated with chronic metabolic disorders, and the assessment of sun protection factor (SPF) as an indicator of potential dermatological and cosmetic applications. Additionally, the study examines analgesic, antimicrobial, and anticancer activities of the extracts against MCF-7 and MDA-MB-231 cell lines. Through this integrated seasonal assessment, the study seeks to identify the periods during which the plant extracts exhibit the highest biological activity, thereby proposing optimal harvesting seasons for the preparation of effective and safe natural formulations for therapeutic and preventive applications.

## 2. Results and Discussion

### 2.1. Total Phenolic and Flavonoid Content

**Figure 1:**
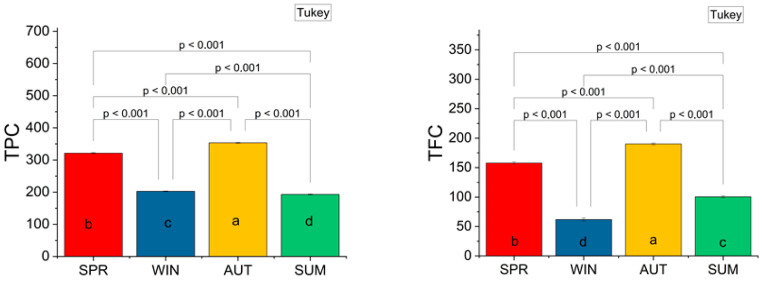
Seasonal variation in total flavonoid content (TFC) and total phenolic content (TPC) in ethanolic extracts of *R. officinalis.* TFC expressed as µg QE/mg extract, TPC expressed as µg GAE/mg extract. Bars indicate mean values ± standard deviation for samples collected in spring (SPR), winter (WIN), autumn (AUT), and summer (SUM). a–d denote significant differences between homogeneous groups (*p* ˂ 0.05) as determined by Tukey’s HSD post hoc test.

The seasonal variation in total phenolic content (TPC) and total flavonoid content (TFC) of *R. officinalis* extracts was statistically significant, as illustrated in Figure 1. The highest TPC was observed in autumn (353.21 ± 4.05 µg GAE/mg extract), followed by spring (321.05 ± 5.44 µg GAE/mg), winter (202.52 ± 1.17 µg GAE/mg), and summer (192.92 ± 3.23 µg GAE/mg), with differences confirmed by Tukey’s post hoc test (*p* < 0.001). The same trend was evident for TFC, which peaked in autumn (190.11 ± 5.65 µg QE/mg extract) and declined through spring (157.66 ± 0.70 µg QE/mg), summer (100.44 ± 0.70 µg QE/mg), and winter (61.88 ± 3.53 µg QE/mg). These data reflect the seasonal influence on phenolic accumulation in *R. officinalis* extract and highlight autumn as the optimal period for harvesting this plant to obtain the highest concentrations of bioactive compounds.

When compared with previously published studies, the values reported here stand among the highest recorded for *R. officinalis*. For example, Hendel et al. [16] documented a phenolic content of 127.1 ± 2.40 µg/mg in methanolic extracts from wild plants in Algeria, a notable level but still markedly lower than the autumn value in this study. Similarly, Yeddes et al. [20] reported a TPC range of 22.91–44.57 mg/g dry weight, while ref. [21] found values between 14.91 and 57.47 mg/g in Kenyan *R. officinalis* samples, reflecting significant geographic variation. Additional reports from Turkey [22] showed phenolic content of 34.1–147.3 µg/mg depending on site and harvest period, while Spanish and Polish samples [23,24] similarly did not reach the peak concentrations measured here. Such variation underscores the interplay between species genetics and environmental factors such as climate, soil conditions, and phenological stage at harvest. The impact of extraction method also cannot be ignored. For instance, Megateli and Krea [25] found that inductive heating boosted TPC to 127.87 ± 2.1 mg GAE/g and TFC to 14.48 ± 1.5 mg QE/g, while Dhouibi et al. [26] reported ultrasonic-assisted extraction (UAE) of *R. officinalis* yielded TPC values from 49.14 to 85 mg/g, higher than those obtained through conventional maceration or Soxhlet methods (13.31–17.32 mg/g). Similarly, Nguyen-Kim et al. [27] obtained a TPC of 197.28 ± 3.11 mg GAE/g under carefully optimized extraction conditions. Nevertheless, despite the demonstrated benefits of advanced extraction, the concentrations measured in this study, especially in autumn, clearly surpass many of these previous reports. This suggests that harvest season can be an even more decisive factor than the extraction technique alone. Balanced moisture, moderate temperatures, and changing photoperiods typical of autumn likely stimulate the phenylpropanoid pathway, leading to enhanced biosynthesis of polyphenols and flavonoids through increased phenylalanine ammonia-lyase (PAL) activity [28,29]. Exposure to moderate abiotic stressors such as fluctuating UV levels may further promote reactive oxygen species (ROS) generation, which acts as a signal to activate plant defense pathways and boost antioxidant compound production [30]. These findings are consistent with observations by Hadi Soltanabad et al. [31], who showed that *R. officinalis* harvested in autumn and winter contained greater amounts of phenolics and flavonoids, driven by subtle regional winters and higher precipitation. Likewise, Kabubii et al. [21] demonstrated that seasonal dynamics and agro-ecological factors significantly modulate TPC and TFC, with dry and wet seasons each favoring different phytochemical trends depending on local climate zones. In line with this, Afshar et al. [32] reported that longer sunlight exposure during warmer seasons increased total flavonoids in *R. officinalis*, while colder periods favored higher antioxidant activity through elevated levels of rosmarinic acid, highlighting that both light duration and temperature shifts shape the seasonal balance of key bioactive compounds. This ecological perspective is reinforced by evidence that *R. officinalis* adjusts its secondary metabolism through a network of protective compounds. For example, carnosic acid, anthocyanins, and soluble sugars can act together as a redox system buffering ROS under changing light and temperature [31], which likely contributes to the higher TPC and TFC observed from late summer into autumn. These results emphasize the practical value of aligning harvests with seasonal metabolic peaks to ensure consistent, high-quality extracts for nutraceutical or pharmaceutical use. Future work combining local climate data, key enzyme assays, and gene expression analysis would help clarify how *R. officinalis* fine-tunes its phytochemistry in response to environmental cues.

### 2.2. Chemical Profiling by LC-ESI-MS/MS

**Figure 2:**
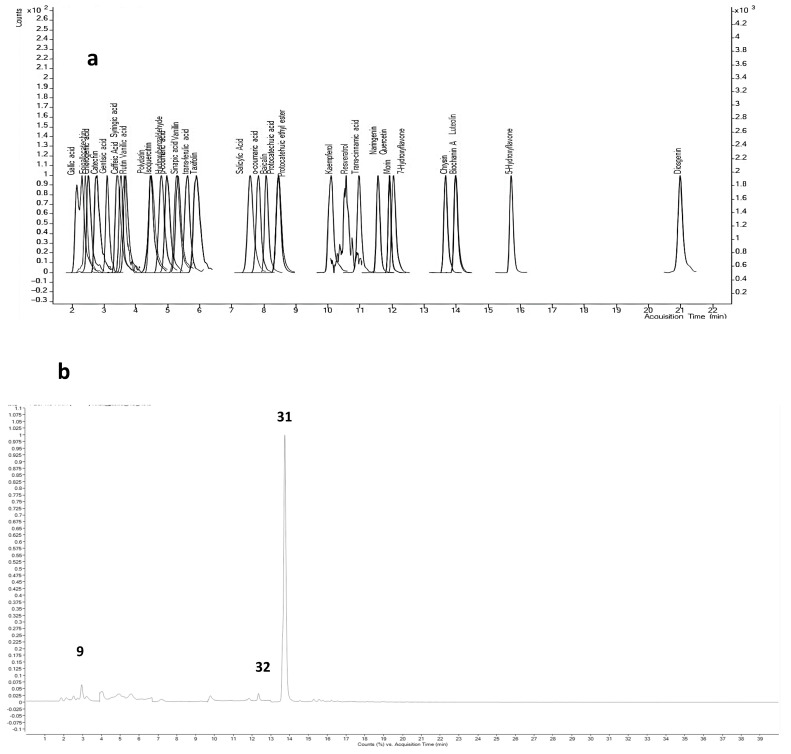

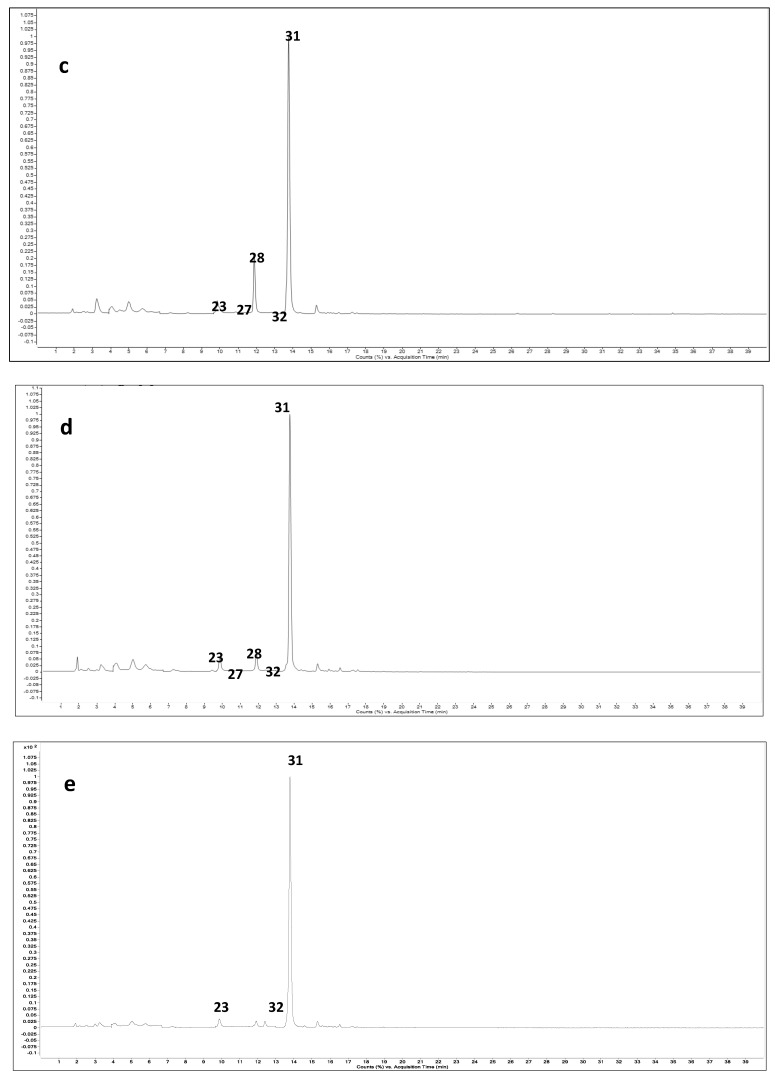
Seasonal LC-ESI-MS/MS chromatograms of *R. officinalis* extracts (**a**) phenolic standards, (**b**) autumn, (**c**) winter, (**d**) spring, (**e**) summer. The numbers indicated above the chromatographic peaks correspond to the compound numbers listed in Table 1. In particular, peak 9 represents rutin, 23 kaempferol, 27 morin, 28 quercetin, 31 luteolin, and 32 biochanin A, which were among the major phenolic and flavonoid constituents detected in the examined rosemary extracts.

LC-ESI-MS/MS analysis was conducted on *R. officinalis* extracts collected in spring, summer, autumn, and winter using HPLC (Agilent 1260 Infinity II) coupled with a mass spectrometer. Identification of compounds was achieved using 34 phenolic standards. The chromatograms of the detected compounds for each extract are presented in Figure 2, and the corresponding data are summarized in Table 1.

Table 1 summarizes the compounds identified in the seasonal extracts of *R. officinalis*. Nineteen compounds were detected in the autumn extract, 17 in both the winter and spring extracts, and 14 in the summer extract.

The results indicate a high content of phenolic compounds, with flavonoids being the major constituents across all seasonal extracts, while other compounds, such as phenolic acids, were detected in low amounts or as traces. Notably, the same key flavonoids biochanin a, luteolin, and kaempferol were consistently identified as the main compounds in the winter, spring, and summer extracts, although their concentrations varied among seasons. In the autumn extract, biochanin a (86.80 µg/g) and luteolin (86.90 µg/g) were predominant, accompanied by rutin (64.05 µg/g). The winter extract also contained biochanin A (82.86 µg/g) and luteolin (38.28 µg/g), with kaempferol (21.65 µg/g) replacing rutin. Similarly, the spring extract was rich in biochanin A (91.37 µg/g), luteolin (15.37 µg/g), and kaempferol (33.75 µg/g). Finally, the summer extract included biochanin A (84.86 µg/g), luteolin (52.32 µg/g), and kaempferol (15.92 µg/g). These findings suggest a stable flavonoid profile throughout the seasons, with quantitative variations likely influenced by seasonal environmental conditions.

Previous studies have determined the chemical profile of *R. officinalis* extracts, highlighting their richness in secondary metabolites such as rosmarinic acid and phenolic diterpenes such as carnosic acid and carnosol responsible for its biological activities [33,34].

Mena et al. [35] reported that *R. officinalis* extract contains flavonoids (mainly flavones, although flavonols and flavanones were also detected), as well as phenolic acids. Among the identified compounds were luteolin-7-O-glucuronide, luteolin-3′-acetyl-*o*-glucuronide, and luteolin. A comparison with our extracts confirms that luteolin is among the major compounds present in all four seasonal extracts.

Velamuri et al. [36] and Francolino et al. [37] reported the presence of polyphenols and flavonoids in *R. officinalis extract*. Among the flavonoids, two luteolin isomers were identified in their study.

Sena et al. [38] reported the identification of various compounds in *R. officinalis* extracts, including flavonoids and phenolic compounds. A comparison with our extracts indicates the presence of several similar compounds, such as caffeic acid and *trans*-ferulic acid in all four seasons, protocatechuic acid in the winter and spring extracts, and *p*-Coumaric acid in the winter season. Additionally, one of our major compounds, luteolin, was also detected.

Based on the comparison between our results and previous studies, we observed that some compounds, such as luteolin, were consistently identified, while the main compounds in our extracts, such as kaempferol, rutin, and biochanin A, were not reported in previous studies. The differences observed between our results and previous studies can be attributed to several factors. Variations in extraction methods (solvent type, extraction time, and conditions), plant part used, geographical origin, and environmental and climatic conditions processing can significantly influence the phenolic composition of plant extracts [8].

### 2.3. Antioxidant Activity Results of R. officinalis Extracts from the Four Seasons

The antioxidant capacity of *R. officinalis* ethanolic extracts exhibited pronounced seasonal variation, underscoring the intricate relationship between environmental factors and secondary metabolite biosynthesis. Among the four seasonal extracts, autumn consistently demonstrated the strongest antioxidant activity (Table 2), exhibiting the lowest IC_50_ values in the DPPH (24.72 ± 0.16 µg/mL), ABTS (14.76 ± 0.60 µg/mL), SNP (17.96 ± 0.44 µg/mL), and superoxide radical scavenging test (ADS, using the alkaline DMSO method) (41.89 ± 0.01 µg/mL) assays. This seasonal superiority coincided with peak levels of total phenolics (TPC) and total flavonoids (TFC), aligning with the central role of these compounds in modulating redox processes via hydrogen atom donation, electron transfer, and free radical stabilization [39,40].

Interestingly, the FRAP assay yielded a divergent pattern, with spring extracts demonstrating the highest iron-reducing capacity (A_0.5_ = 7.62 ± 0.30 µg/mL), suggesting enrichment in compounds with strong electron-donating capacity independent of total phenolic abundance. This divergence underscores the mechanistic heterogeneity of antioxidant assays. While DPPH, ABTS, SNP, and ADS primarily measure radical scavenging through hydrogen atom or single electron transfer, FRAP assesses the ability to reduce Fe^3+^ to Fe^2+^, favoring compounds with pronounced electron-donating capability [41]. The spring extract may thus be enriched in specific reducing agents such as caffeic acid derivatives, glycosylated flavonoids, or even transition-metal chelating terpenes, which are less abundant in other seasons.

**Figure 3:**
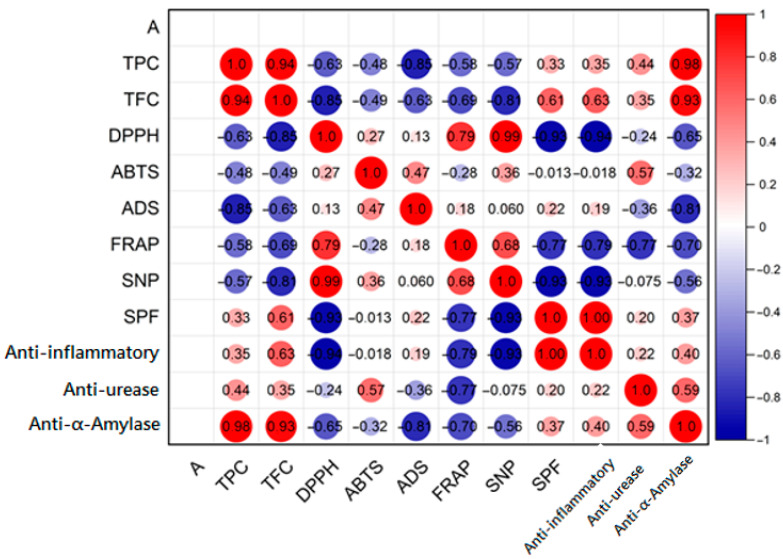
Correlation matrix of bioactivities and phytochemicals in seasonal *R. officinalis* extracts.

Although phenolic and flavonoid contents peaked in autumn alongside elevated antioxidant activity, correlation analysis revealed no statistically significant relationships (*p* > 0.05), as shown in Figure 3. This underscores that antioxidant efficacy depends more on the structural and electronic properties of individual compounds than on total abundance. Key molecular factors, such as bond dissociation enthalpy, ionization potential, and proton affinity, govern the capacity of phenolics to donate electrons or hydrogen atoms [42,43]. Structure–activity relationship (SAR) and DFT modeling studies confirm that ortho-dihydroxy substitution and resonance stabilization significantly enhance antioxidant potential [44]. Additionally, refs. [45,46] showed that antioxidant performance often reflects the interplay of multiple compound classes, which no single assay can fully capture. Thus, the lack of correlation likely stems from qualitative compositional differences and synergistic effects among phenolics, flavonoids, and terpenoids in *R. officinalis*. Future studies should integrate advanced metabolomics with reactivity modeling to identify the structural determinants of seasonal antioxidant activity.

From an ecological perspective, environmental stressors such as cooler temperatures, shorter photoperiods, and elevated oxidative pressures in autumn and winter can upregulate phenylpropanoid biosynthetic enzymes such as phenylalanine ammonia-lyase (PAL) and chalcone synthase (CHS). This biochemical response stimulates the accumulation of redox-active compounds, supporting our findings of enhanced antioxidant activity during these seasons [47,48].

These patterns are supported by Hadi Soltanabad et al. [31], who reported peak flavonoid levels in autumn and higher carnosic acid and total phenolic concentrations in winter in Iranian *R. officinalis*. Interestingly, their DPPH results showed minimal seasonal variation, reinforcing the importance of compound identity over quantity.

Further regional confirmation comes from Kabubii et al. [21], who evaluated *R. officinalis* from various agro-ecological zones in Kenya. While DPPH activity did not vary significantly between wet and dry seasons, FRAP values were consistently higher in the dry season. Such assay-specific differences reflect the diversity of antioxidant mechanisms, with DPPH assessing hydrogen atom donation and FRAP measuring electron transfer to metal ions [41].

In contrast, Yeddes et al. [20] showed a comprehensive analysis of *R. officinalis* extracts from Tunisia, showing that both bioclimatic region and season significantly influenced antioxidant behavior. The study reported the highest DPPH scavenging activity during summer, correlating strongly with increased levels of total phenolics, flavonoids, and carnosic acid. Interestingly, the highest FRAP and ABTS activities occurred in winter, coinciding with elevated rosmarinic acid concentrations, suggesting that different compounds dominate activity in different seasons and assays. Their findings highlight that antioxidant behavior is governed not by bulk content only, but by compositional richness and compound-specific efficacy, echoing our conclusion that no single assay can fully represent antioxidant capacity [49].

Notably, the IC_50_ value of our winter extract (114.42 ± 4.38 µg/mL) aligns closely with findings from previously published research by Al-jaafreh [50], who reported a similar DPPH activity (106.64 ± 6.44 µg/mL) for *R. officinalis* harvested in May in southern Jordan. Conversely, the strong activity of our autumn extract (24.72 ± 0.16 µg/mL) is comparable to that reported by Bendif et al. [51] for samples collected in early spring in Algeria (25.6 ± 0.4 µg/mL), suggesting that regional climatic similarities rather than strict calendar seasons may shape the phenolic biosynthetic responses that underpin antioxidant activity.

Beyond environmental influence, methodological factors also play a crucial role in shaping observed antioxidant efficacy. For instance, Megateli and Krea [25] employed electromagnetic induction heating (EMIH) and reported extraordinarily potent DPPH inhibition (IC_50_ = 0.00148 µg/mL), demonstrating that thermal-assisted extraction can yield significantly more reactive phenolic profiles. Similarly, Dhouibi et al. [26] showed that ultrasound-assisted extraction (UAE) dramatically improved antioxidant performance (IC_50_ = 0.13 mg/mL), outperforming both supercritical fluid and combined UAE–SFE techniques. Furthermore, Nguyen-Kim et al. [27] reported a notable IC_50_ of 9.4 ± 0.1 µg/mL, reinforcing that both chemotype variability and extraction conditions critically influence the final antioxidant profile. These findings underscore that comparisons across studies must consider not only geographic and seasonal contexts but also the extraction methodologies and compound-specific enrichment.

Collectively, our findings highlight autumn as the optimal season for harvesting *R. officinalis* to obtain extracts with superior antioxidant potential, likely due to seasonal upregulation of phenylpropanoid pathways and favorable accumulation of redox-active compounds. However, other seasons may offer distinct therapeutic benefits, and may provide different phytochemical profiles that could be beneficial for other applications, suggesting a complex interplay of seasonal effects on *R. officinalis*’s bioactivity.

### 2.4. α-Amylase Inhibitory Activity of R. officinalis Extracts

The inhibitory potential of *R. officinalis* ethanolic extracts harvested across four seasons was assessed against two enzymes of pharmacological interest. α-Amylase, a key enzyme in carbohydrate digestion, contributes to postprandial hyperglycemia and is thus a target for managing type 2 diabetes

The ethanolic extracts of *R. officinalis* exhibited distinct seasonal variation in α-amylase inhibitory activity. The autumn and spring extracts demonstrated measurable inhibition, with IC_50_ values of 1142.04 ± 11.32 µg/mL and 1171.73 ± 19.25 µg/mL, respectively, while the winter and summer extracts showed no detectable activity within the tested concentration range (IC_50_ > 1600 µg/mL) (Table 3). These patterns emphasize the selective distribution and seasonal regulation of bioactive metabolites that modulate enzyme activity, in alignment with both the metabolic rhythms of the plant and external environmental cues.

Although the α-amylase inhibition observed in our extracts appears moderate, the IC_50_ values are considerably lower than those of the synthetic standard acarbose (3650.93 ± 10.70 µg/mL), indicating higher inhibitory potency under the current assay conditions. The inhibition likely arises from synergistic interactions among multiple phenolic and diterpenoid compounds such as rosmarinic acid, carnosic acid, and apigenin derivatives, rather than a single dominant molecule [52,53]. These compounds, structurally characterized by hydroxylated aromatic systems and conjugated moieties, are known to bind α-amylase through hydrogen bonding and π-π stacking at catalytic or peripheral sites, thereby interfering with substrate access and enzyme functionality [54].

Although direct assessments of seasonal variation in α-amylase inhibition for *R. officinalis* are scarce, extensive phytochemical profiling reveals that compounds responsible for this activity, particularly rosmarinic and carnosic acids, reach their peak abundance in the cooler, wetter months of autumn and winter [31]. This accumulation is mainly driven by environmental cues like low temperature and high humidity. Our correlation matrix (Figure 3) revealed strong negative correlations between α-amylase IC_50_ values and both total phenolic (r = −0.99) and flavonoid content (r = −0.94), supporting the mechanistic link between polyphenol abundance and enzymatic inhibition. Although the winter extract had high phytochemical content, it showed no inhibitory effect, a discrepancy that may stem from post-harvest degradation, bioavailability issues, or co-extracted antagonistic constituents.

In contrast Bejenaru et al. [55] observed peak rosmarinic acid concentrations in winter, while [56] reported high carnosic acid and antioxidant activity in summer-collected extracts. This discrepancy likely reflects differences in environmental conditions, plant chemotypes, or post-harvest processing, underscoring the complex interplay between climate-driven biosynthesis and phytochemical stability. In our study, the autumn extract, which also exhibited the highest antioxidant activity, demonstrated the most potent α-amylase inhibition, reinforcing a functional link between antioxidant capacity and enzyme-modulating potential. This finding is consistent with previous reports in related Lamiaceae species, where elevated antioxidant levels were associated with enhanced antidiabetic activity [57,58].

Discrepancies between our findings and published data further underscore the role of methodological variables. For instance, Pınar Ercan and Sedef Nehir El [59] reported a much stronger α-amylase inhibitory effect (IC_50_ = 95.65 ± 2.73 µg/mL) using Turkish *R. officinalis* extracts, and ref. [60] recorded inhibition above 90% in summer-harvested Jordanian samples. In contrast, our summer extract exhibited no activity. Such inconsistencies may be attributed to differences in extraction protocols (solvent polarity, maceration duration, temperature), cultivar or chemotype, harvest maturity, drying procedures, or enzyme assay calibration. Similarly, phytochemical degradation during post-harvest processing can alter the bioactivity profile, particularly for heat- or light-sensitive phenolic acids.

Taken together, these results demonstrate that seasonal variation significantly influences the enzyme-inhibitory capacity of *R. officinalis*, likely through fluctuations in the biosynthesis and accumulation of structurally active phenolics and terpenoids. Spring and autumn emerge as the most promising harvest periods for maximizing α-amylase inhibition, offering potential for the development of plant-based therapeutics targeting postprandial glycemic control. These findings not only illuminate the phytochemical underpinnings of enzyme inhibition but also position *R. officinalis* as a viable candidate for functional food or nutraceutical development tailored by seasonal optimization.

### 2.5. Urease Inhibitory Activity of R. officinalis Extracts

Urease is a clinically significant enzyme that catalyzes the conversion of urea into ammonia and is implicated in the development of several pathological conditions, including *Helicobacter pylori*-associated gastric ulcers, hepatic encephalopathy, and urinary tract infections. Consequently, increasing attention has been directed toward the identification of effective urease inhibitors from natural sources as promising alternatives to conventional synthetic agents.

Among the four seasonal ethanolic extracts of *R. officinalis*, urease inhibitory activity was observed exclusively in the spring sample, which demonstrated an IC_50_ of 62.26 ± 0.58 µg/mL (Table 4). All other extracts (autumn, summer, and winter) exhibited no detectable inhibition under the tested conditions. This selective activity pattern strongly suggests that the presence and relative abundance of specific phytochemical constituents, rather than total phenolic or flavonoid content alone, govern the observed enzyme inhibition.

Despite not exhibiting the highest total phenolic or flavonoid content, the spring extract demonstrated selective urease inhibition, suggesting that activity stems from specific phytochemical constituents rather than overall phenolic abundance. The absence of a statistically significant correlation between urease inhibition and TPC or TFC supports this interpretation (Figure 3). Since the present study did not involve individual compound identification, direct attribution of activity to specific molecules remains speculative. However, previous phytochemical investigations of seasonal variation in *R. officinalis* provide valuable contextual insight. In particular, ref. [55] reported that caffeic acid, a hydroxycinnamic acid known to inhibit urease, reaches its maximal concentration in *R. officinalis* during spring. Mechanistic studies in other species have confirmed non-competitive inhibition by caffeic acid, along with chlorogenic acid, and luteolin, mediated through nickel chelation at the active site and allosteric disruption of catalytic conformations [61].

The selective urease inhibition observed in the spring extract, despite higher TPC values in autumn, underscores that bioactivity is governed by phytochemical composition rather than total phenolic content. Seasonal variations in light, temperature, and developmental stage influence secondary metabolism, potentially promoting the spring-specific accumulation of labile urease-active compounds [62,63]. The absence of activity in other seasons, notably autumn, suggests that molecular specificity, such as the presence of ortho-dihydroxylated phenolics or terpene-based chelators, is more critical than quantity.

Although no previous studies have explicitly linked urease inhibition to spring-harvested plant extracts, the findings here suggest a previously unrecognized seasonal window for maximizing enzyme-inhibitory potential in *R. officinalis*. Urease inhibition has been reported in other Lamiaceae species like *Salvia officinalis* and *Mentha pulegium* [64,65]. However, these studies lack seasonal resolution. Thus, our data provide novel evidence that spring harvest may optimize urease-inhibitory profiles in *R. officinalis*, warranting further bioactivity-guided fractionation and kinetic modeling to identify the responsible constituents and determine the mode of inhibition.

### 2.6. Sun Protection Factor (SPF)

The SPF values of *R. officinalis* extracts collected during different seasons were evaluated by measuring their ultraviolet (UV) absorbance within the 290–320 nm wavelength range. The corresponding results are summarized in Table 5.

All extracts demonstrated an effective ultraviolet (UV) absorption capacity, with SPF values ranging from 25.18 to 32.46, which are substantially higher than the recommended minimum reference value for UV protection (SPF ≥ 6) according to Takayama et al. [66]. The summer extract exhibited the highest SPF value (32.46 ± 0.35), followed by the autumn (30.97 ± 0.42) and spring extracts (30.79 ± 0.52), while the winter extract showed the lowest value (25.18 ± 0.32).

Interestingly, these seasonal differences in SPF did not align with total phenolic (TPC) or flavonoid content (TFC), as no statistically significant correlations were observed (Figure 3). Autumn and spring extracts displayed the highest TPC and TFC values, yet the summer extract demonstrated the highest SPF. This dissociation underscores that photoprotective efficacy is not solely determined by total compound abundance but rather by the specific molecular structures, UV-absorbing chromophores, and synergistic interactions among bioactive constituents [67].

However, SPF showed exceptionally strong negative correlations with DPPH (r = −0.93) and SNP (r = −0.93), suggesting that antioxidant capacity—particularly radical scavenging—is closely associated with photoprotective potential. This observation is mechanistically plausible, as UV radiation induces reactive oxygen species (ROS) in skin, triggering oxidative stress, photoaging, and inflammation. Therefore, compounds that neutralize ROS contribute indirectly to SPF enhancement by mitigating UV-induced cellular damage [67,68].

Although individual phytochemicals were not identified in the present study, it is well-established that *R. officinalis* accumulates various flavones and conjugated phenolic acids with strong UV-absorbing capacity, particularly under high light and temperature conditions typical of summer. These include compounds such as luteolin, apigenin, and caffeic acid derivatives, whose conjugated double bonds and aromatic systems efficiently absorb UVB wavelengths [69,70].

These results collectively emphasize that *R. officinalis* extracts, particularly those harvested in summer and autumn, are promising candidates for the development of natural sunscreen formulations.

### 2.7. Antimicrobial Activity Study of Ethanolic Extracts of R. officinalis

The antimicrobial activity of ethanolic extracts of *R. officinalis*, collected during the four seasons, was assessed at different concentrations. The corresponding results are summarized in Table 6.

This study examined the effect of harvest season on the antibacterial activity of ethanolic *R. officinalis* extracts. Overall, antibacterial activity was weak across all seasons and tested microorganisms. *Staphylococcus aureus* ATCC 25932 was the only strain exhibiting measurable sensitivity, with the autumn extract producing the highest inhibition zone (13 mm at 80 mg/mL), compared with 7–9 mm for extracts obtained in winter, spring, and summer. No inhibitory activity was observed against *Escherichia coli*, *Pseudomonas aeruginosa*, or *Candida albicans*, and only minimal inhibition of *Bacillus subtilis* was detected in the autumn sample. These results indicate that seasonal variation exerts only a minor influence on the antibacterial efficacy of ethanolic *R. officinalis* extracts.

The slightly enhanced activity observed in autumn suggests limited seasonal modulation; however, the magnitude of this effect was small and not dose-dependent. The 4 mm increase in inhibition zone diameter against *S. aureus* remains far below the activity of the gentamicin control and is unlikely to be biologically or practically significant. Seasonal differences in extract composition were observed, but no clear correlation was identified between individual phenolic constituents and antibacterial activity, indicating that seasonal phytochemical variation alone does not predict antimicrobial potency.

The selective susceptibility of *S. aureus* is consistent with the greater permeability of Gram-positive cell walls to polar compounds, whereas the resistance of Gram-negative bacteria and yeast reflects the protective role of outer membranes and complex cell envelopes [71]. The weak activity observed aligns with previous reports showing that polar *R. officinalis* extracts generally exhibit limited antimicrobial efficacy compared with essential oils or non-polar fractions [72].

The limited antimicrobial activity may also be attributed to the concentration range tested (10–80 mg/mL). Previous studies have demonstrated that *R. officinalis* ethanolic extracts typically require concentrations exceeding 100–200 mg/mL to achieve significant inhibitory effects against resistant bacterial strains, particularly Gram-negative species [71,73]. No antimicrobial activity against *E. coli*, *Salmonella*, and other Gram-negative bacteria has been reported, even at concentrations up to 100 mg/mL, while concentrations of 200 mg/mL have been employed to control bacterial biofilms effectively [71,73]. Furthermore, Gram-negative bacteria have been shown to require minimum inhibitory concentration (MIC) values of 60 mg/mL or higher, substantially greater than the 2–15 mg/mL needed for Gram-positive strains [31]. These findings suggest that higher concentrations may be necessary to overcome the protective barriers of bacterial outer membranes and achieve therapeutically relevant activity. Future investigations should explore higher concentration ranges (100–250 mg/mL) and alternative extraction methodologies to fully assess the antimicrobial potential of seasonal *R. officinalis* extracts.

In conclusion, although autumn harvesting resulted in marginally higher activity, ethanolic *R. officinalis* extracts showed insufficient and narrow-spectrum antibacterial effects. Seasonal optimization alone is therefore inadequate to confer meaningful antimicrobial efficacy. The marginally higher autumn activity may reflect seasonal variation in phenolic content. Autumn environmental conditions, moderate temperatures, reduced water stress, and declining photoperiods can stimulate the phenylpropanoid pathway, potentially increasing rosmarinic acid and flavonoid concentrations [74,75]. However, the observed differences remain small and of questionable practical significance. The selective, weak response against *S. aureus* likely reflects the greater permeability of Gram-positive peptidoglycan cell walls compared to the lipopolysaccharide-rich outer membrane of Gram-negative bacteria, which effectively excludes polar compounds [71,76,77]. Fungal resistance further demonstrates the limited spectrum of ethanolic extracts.

### 2.8. Anti-Inflammatory Activity Results of the Seasonal Extracts of R. officinalis

#### 2.8.1. In Vitro Evaluation of Anti-Inflammatory Activity

The anti-inflammatory potential of *R. officinalis* extracts was assessed using the protein denaturation inhibition assay, a well-established model for screening bioactive compounds that stabilize protein structure under thermal stress [78]. Denaturation, driven by the disruption of non-covalent interactions and disulfide bridges, leads to protein dysfunction and is implicated in inflammatory processes [79]. Plant-derived compounds, particularly phenolic acids and flavonoids, are known to prevent such changes and thus serve as promising candidates for natural anti-inflammatory agents [80].

In the present study, ethanolic extracts of *R. officinalis* collected during four seasons were tested and compared to diclofenac sodium, a standard non-steroidal anti-inflammatory drug (NSAID). The anti-inflammatory activity was expressed as the concentration required to inhibit 50% of protein denaturation (IC_50_), and results are presented in Table 7.

The winter extract exhibited the strongest anti-inflammatory activity, recording the lowest IC_50_ value (28.60 ± 2.84 µg/mL), surpassing even the reference drug diclofenac sodium (40.90 ± 0.89 µg/mL). This suggests the presence of potent phytochemicals in the winter extract that may, in certain cases, outperform conventional non-steroidal anti-inflammatory drugs (NSAIDs). In contrast, the summer extract showed the weakest activity, with the highest IC_50_ value (125.61 ± 1.22 µg/mL), while the values recorded in spring and autumn were similar (106.01 ± 6.17 and 107.78 ± 2.09 µg/mL, respectively).

These findings are consistent with the study by Abdulbary et al. [81], which highlighted the ability of *R. officinalis* extracts to reduce heat-induced hemolysis, indicating a membrane-stabilizing effect on erythrocytes. This effect is believed to be attributed to the presence of active compounds such as tannins, which play an important role in stabilizing lysosomal and erythrocyte membranes by binding to Ca^2+^ and Mg^2+^ ions [82]. Furthermore, the inhibition of protein denaturation may be associated with flavonoids and phenolic acids known for their anti-inflammatory properties [80].

These results suggest that plant extracts, particularly those obtained during the winter season, may serve as promising natural alternatives to conventional anti-inflammatory drugs, with potentially fewer side effects.

Interestingly, no statistically significant correlation was found between IC_50_ values and either total phenolic content (TPC) or total flavonoid content (TFC) (Figure 3)**;** these results imply that the anti-inflammatory effectiveness of the extracts may not solely depend on the total quantities of phenolics or flavonoids but is likely influenced by the qualitative composition and structure of these compounds, as well as their synergistic interactions with other bioactive constituents, particularly terpenoids, which are well known to be abundant in *R. officinalis* extracts. Similarly, the summer extract (RSummer) exhibited the lowest anti-inflammatory activity (IC_50_ = 125.61 ± 1.22 µg/mL), which coincided with the lowest phenolic content (192.92 ± 3.23 µg/mg), reinforcing the hypothesis of a qualitative rather than purely quantitative relationship between phytochemical composition and biological activity.

Therefore, this study underscores that quantitative analysis of phenolics and flavonoids alone is insufficient to predict anti-inflammatory potential. A comprehensive qualitative analysis is essential to identify the bioactive constituents responsible for the seasonal variations in anti-inflammatory efficacy.

#### 2.8.2. In Vivo Anti-Inflammatory Activity of the Winter Extract of *R. officinalis*

Although the spring extract exhibited the highest phenolic and flavonoid contents, the winter extract showed superior in vitro anti-inflammatory activity. Therefore, the winter extract was selected for in vivo evaluation following a bioactivity-guided approach, ensuring the use of the most promising candidate while minimizing animal use.

In this study, the potential anti-inflammatory effects of *R. officinalis* extracts were assessed using preclinical in vivo models. Whole *R. officinalis* extracts are more commonly employed than isolated compounds, with maceration being the predominant extraction methods. Administration routes included intraperitoneal (IP) and oral, with doses adjusted according to the method used. Treatments were given either as a single dose or daily for an average of 21 days, often starting prior to inflammation induction. Commonly assessed biomarkers included tumor necrosis factor-alpha (TNF-α), interleukins IL-1β, IL-6, and IL-10, as well as myeloperoxidase (MPO), catalase (CAT), glutathione (GSH), glutathione peroxidase (GPx), malondialdehyde (MDA), and superoxide dismutase (SOD). Optimal outcomes have been reported with a 60 mg/kg IP dose of carnosic acid, a 400 mg/kg oral dose of *R. officinalis*, and a 10 mg/kg IP dose of rosmarinic acid [83].

The in vivo anti-inflammatory activity of *R. officinalis* winter extract was evaluated using the carrageenan-induced paw edema model in Swiss albino mice, a widely accepted method for assessing acute inflammatory responses. Carrageenan injection induced significant edema in the control group (51.80 ± 1.92%), confirming the inflammatory effect of the model. Treatment with diclofenac reduced paw thickness to 14.00 ± 1.58%, corresponding to 72.97% inhibition. The winter extract produced a dose-dependent anti-inflammatory response, with the 500 mg/kg dose achieving 62.93% inhibition and the 100 mg/kg dose showing 55.98% inhibition (Table 8). These results are consistent with the extract’s in vitro activity, highlighting its potential as an effective anti-inflammatory agent.

These findings demonstrate that the winter extract of *R. officinalis* exhibits substantial in vivo anti-inflammatory activity, approaching the efficacy of diclofenac at higher doses. This supports earlier observations by Grigore et al. [84], who reported 50.39% edema inhibition with *R. officinalis* extract, comparable to aspirin (54.40% at 100 mg/kg). The current results further emphasize the therapeutic relevance of seasonally optimized extracts, positioning winter-harvested *R. officinalis* as a promising natural anti-inflammatory candidate with potentially fewer adverse effects than conventional NSAIDs.

### 2.9. Analgesic Activity of the Winter Extract of R. officinalis

Numerous plant-derived extracts possess notable analgesic activity due to their phytoconstituents with anti-inflammatory properties and the ability to modulate nociceptive signaling pathways [85]. Among the key factors influencing such pharmacological effects is the season of harvest, which affects the biosynthesis and accumulation of bioactive secondary metabolites. To investigate this, the winter ethanolic extract of *R. officinalis* was selected for the analgesic test based on its superior in vitro anti-inflammatory activity (IC_50_ = 28.60 µg/mL), non-toxic profile in acute toxicity studies (2000 mg/kg), and favorable phytochemical composition. This choice aligns with ethical principles to minimize animal use by testing the most promising extract only.

Treatment with *R. officinalis* winter extract led to a dose-dependent reduction in the number of abdominal writhes. At 500 mg/kg, the extract provided 68.41% inhibition, nearly equivalent to that of paracetamol (70.43%). Even at 100 mg/kg, it achieved a substantial 57.68% inhibition, highlighting its potent peripheral analgesic effect (Table 9). The data suggest that the analgesic activity of *R. officinalis* is likely mediated through suppression of inflammatory mediators involved in acetic acid-induced nociception, such as prostaglandins and bradykinin [85].

These results are in line with those reported by [86], who observed a pain inhibition percentage of 28% with *R. officinalis* extracts compared to 57% with indomethacin, emphasizing the variation in efficacy across experimental conditions and extract composition. Collectively, these findings underscore the promising analgesic properties of winter-harvested *R. officinalis*, supporting its use as a natural alternative to conventional analgesics.

### 2.10. Acute Toxicity

No mortality was observed in rats administered increasing doses of the winter ethanolic extract of *R. officinalis*, even at the highest tested dose of 2000 mg/kg body weight. Throughout the 14-day observation period, the animals showed no signs of behavioral or clinical toxicity. Specifically, there were no indications of hyperactivity, ataxia, tremors, convulsions, salivation, diarrhea, lethargy, sleepiness, or coma. The rats remained physically active and maintained normal behavior and physiological functions. These findings suggest that the winter extract is well tolerated and does not exhibit any apparent acute toxic effects at a dose of 2000 mg/kg. Therefore, the winter extract of *R. officinalis* can be considered non-toxic at this dosage level.

### 2.11. Anticancer Effects of Seasonal R. officinalis Extracts on Breast Cancer Cell Lines

The cytotoxic effects of *R. officinalis* were evaluated on both MCF-7 and MDA-MB-231 breast cancer cell lines, and IC_50_ values were determined accordingly. A range of concentrations—200, 100, 50, 25, 12.5, and 6.25 µg/mL—was tested. Wells treated with only the culture medium, without the extract, served as the negative control.

The IC_50_ values of *R. officinalis* for both cell lines were found to be relatively high (Table 10), indicating a moderate cytotoxic effect. Although no significant seasonal variation was observed, IC_50_ values were generally higher in extracts obtained during autumn and winter compared to those from spring and summer.

In the MDA-MB-231 cell line, the spring extract exhibited notably lower IC_50_ values than those of other seasonal extracts tested on the same cell line.

In addition, these values were also lower than all seasonal extracts tested on MCF-7. This suggests a potentially higher efficacy of the spring-harvested *R. officinalis* against triple-negative breast cancer cells. Given the clinical challenges associated with hormone-independent breast cancers, the observed seasonal variation, particularly the spring extract’s enhanced activity on MDA-MB-231 cells, warrants further investigation.

In a study comparable to ours, ref. [87] likewise reported lower IC_50_ values in MDA-MB-231 cells than in MCF-7 cells, indicating greater sensitivity of the triple-negative line. However, the IC_50_ values in that report were generally lower than those observed here, a difference that may reflect methodological factors, particularly our initial seeding density of 1.5 × 10^4^ cells. Moreover, unlike our work, that study did not evaluate seasonal variation in *R. officinalis*, which is a dimension our study explicitly addresses.

### 2.12. Chemometric Analysis

Chemometric methods provide information about the similarity of measured parameters between different sampling locations. For interpreting analytical results, different chemometric methods are employed, such as principal component analysis (PCA), cluster analysis (CA), and linear discriminant analysis (LDA) [88].

**Figure 4:**
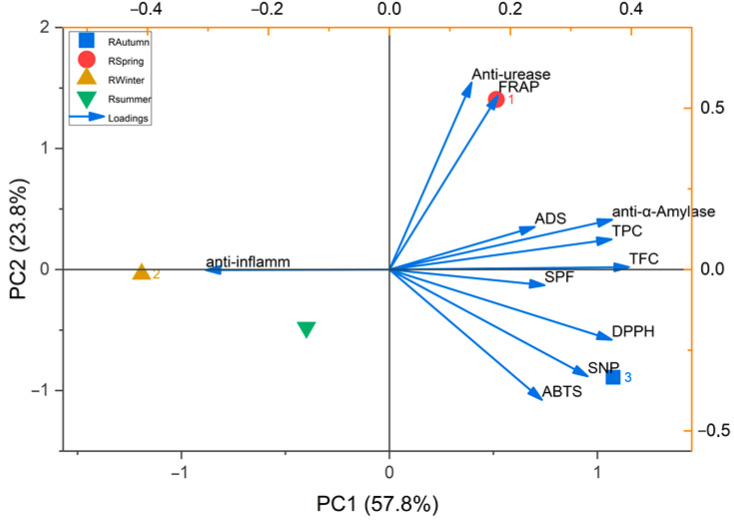
Principal component analysis of seasonal *R. officinalis* extracts and associated bioactivities.

The PCA performed on the dataset characterizing the biological potential and phenolic content of the examined *R. officinalis* extracts suggested that the first two principal components describe more than 81% of the samples’ variability. Figure 4 illustrates the clustering of *R. officinalis* extracts into four distinct groups based on their bioactive properties. Group 1, represented by the spring extract and positioned in the positive region of the second principal component (PC2), is characterized by notable anti-urease activity and strong antioxidant potential as measured by the FRAP assay. The winter extract forms the second group, located in the negative region of the first principal component (PC1), and is distinguished by its high anti-inflammatory activity. The positive region of PC1 is occupied by the autumn extract, which constitutes the third group. This extract is notable for its high phenolic and flavonoid content, as well as strong α-amylase inhibitory activity and significant antioxidant effects, as assessed by the DPPH, ABTS, and SNP methods. Finally, the summer extract forms the fourth group in the third principal component (PC3), accounting for over 18% of the total variability among samples. This extract is primarily characterized by its elevated sun protection factor (SPF) potential and relatively low antioxidant defense activity.

## 3. Materials and Methods

### 3.1. Chemicals and Reagents

All solvents used in this study were of analytical grade and sourced from Honeywell (Charlotte, NC, USA). The following reagents were utilized: 1,1-diphenyl-2-picrylhydrazyl (DPPH), Folin–Ciocalteu reagent, aluminum chloride hexahydrate (AlCl_3_ 6H_2_O), sodium carbonate (Na_2_CO_3_), ferrozine, potassium ferricyanide, ammonium molybdate ((NH_4_)_6_Mo_7_O_24_·4H_2_O), 2,2′-azinobis(3-ethylbenzothiazoline-6-sulfonic acid) diammonium salt (ABTS^●+^), sodium molybdate (Na_2_MoO_4_), and sodium acetate (C_2_H_3_NaO_2_), all purchased from Sigma-Aldrich GmbH (Steinheim, Germany). Reference compounds were obtained from EXTRASYNTHESE (Genay Cedex, France), including quercetin (C_15_H_10_O_7_), butylated hydroxytoluene (BHT, C_15_H_24_O), gallic acid (C_11_H_16_O_2_), butylated hydroxyanisole (BHA, C_11_H_16_O_2_) ascorbic acid (C_6_H_8_O_6_), EDTA, and α-tocopherol.

### 3.2. Collection, Preparation, and Extraction of Plant Material

The aerial parts of *R. officinalis* were collected during the 2022/2023 period, with sampling conducted at the mid-point of each season (autumn, winter, spring, and summer). The plant species was taxonomically authenticated by the Department of Chemistry, University of El Oued, Algeria, and a voucher specimen was deposited in the Herbarium of the Laboratory of Biomass, ENS-Kouba, Algiers, Algeria, under the code Ros-offc-001-4-2022. Healthy, mature plants growing under similar environmental conditions were selected from the El Kef Lakhdar area in Medea province, located at 1164 m above sea level (35°57′33″ N, 3°12′16″ E).

Upon collection, plant materials were transported in clean, airtight containers to prevent degradation and contamination. The samples were thoroughly washed with deionized water to remove impurities, then spread on clean trays and air-dried in the shade at 20–25 °C for 15 days to preserve thermolabile and photosensitive compounds. Once dried, the plant parts (flowers, leaves, and stems) were finely ground using an agate mortar and pestle or an electric grinder to achieve a uniform particle size (<200 µm). The powdered material was stored in airtight containers in a cool, dry, and dark place until further processing.

The extraction of bioactive compounds from *R. officinalis* aerial parts was performed using a maceration method with hydroethanolic solvent. Fifteen grams of dried, finely ground plant material were macerated in 500 mL of ethanol-distilled water mixture (80:20, *v*/*v*) at room temperature for 24 h under continuous mechanical stirring. After the initial maceration period, the mixture was then filtered. To ensure complete extraction of phytochemical compounds, this extraction process was repeated three times using fresh solvent on the same plant material.

The filtrates from all three extraction cycles were pooled and concentrated using a rotary evaporator (Buchi R-210 Rotavapor, Manufacturer, Bochnia, Poland) at 40 °C under reduced pressure to remove the solvent completely. The final extracts were stored in amber glass bottles in a refrigerator at 4 °C until ready for analysis. The percentage of yield of crude extract was calculated using the following Equation (1):

% Yield = (Weight of dried crude extract (g)/Weight of dried plant sample (g)) × 100(1)

### 3.3. Total Secondary Metabolites Content

#### 3.3.1. Total Phenolic Content (TPC)

Total phenolic content was determined using the Folin–Ciocalteu colorimetric method adapted for a microplate format [89]. Briefly, 20 µL of the extract (1 mg of the extract was dissolved in methanol) was mixed with 100 µL of diluted Folin–Ciocalteu reagent (1:10 *v*/*v* with distilled water) and 75 µL of 7.5% (*w*/*v*) sodium carbonate solution in each well. The mixture was incubated at room temperature in the dark for 2 h. Absorbance was measured at 765 nm using a microplate reader PerkinElmer-Multimode. Gallic acid was used to construct a standard calibration curve, and results were expressed as µg gallic acid equivalents per mg dry extract (µg GAE/mg). All measurements were performed in triplicate.

#### 3.3.2. Total Flavonoid Content (TFC)

Total flavonoid content was determined using the aluminum chloride colorimetric method [90]. A mixture of 50 µL extract, 130 µL methanol, 10 µL potassium acetate (1 M), and 10 µL Al(NO_3_)_3_ (10%) was incubated in the dark for 40 min, and absorbance was measured at 415 nm. Results were expressed as µg quercetin equivalents per mg of extract (µg QE/mg).

### 3.4. Chemical Profiling Analysis

The chemical profiling of *R. officinalis* extracts collected in spring, summer, autumn, and winter was carried out using LC-ESI-MS/MS analysis, following the method previously described [91]. The analysis employed 34 standards using an HPLC system coupled to a mass spectrometer, with ionization performed via electrospray ionization (ESI). Chromatographic separation was achieved on a Poroshell 120 EC-C_18_ column (100 mm × 4.6 mm, 2.7 µm particle size). For sample preparation, 50 mg of crude extract was accurately weighed and dissolved in 1 mL of a mixed solvent system composed of acetonitrile–methanol–water (1:1:1, *v*/*v*/*v*). The mixture was vortexed until complete dissolution and sonicated if necessary. Liquid–liquid extraction was then performed by adding 0.8 mL of hexane, followed by centrifugation at 7000 rpm for 5 min. The lower phase was collected and diluted (1:4, *v*/*v*). Prior to analysis, samples were filtered through a 0.25 µm syringe filter. Analysis was performed using a 4 µL injection volume of extract, in full loop injection mode. A flow rate of 0.4 mL/min was maintained using a gradient elution system. The mobile phase consisted of ultrapure water and ethanol containing 0.1% formic acid and 5 mM ammonium. The gradient started at 15.12% methanol for 1 min, increased to 50% over 30 min, then to 90% over the next 30 min, and finally returned to 10% at 32 min for column re-equilibration.

### 3.5. Antioxidant Activity Assays

#### 3.5.1. DPPH Radical Scavenging

The ability of the extracts to scavenge DPPH free radicals was assessed using Blois’ method [92] with minor modifications. In a 96-well microplate, 160 µL of a freshly prepared 0.1 mM DPPH solution in ethanol was combined with 40 µL of sample (having a concentration rage from 12.5 to 800 µg/mL) or standard antioxidant (BHA and ascorbic acid). The reaction mixtures were incubated in the dark at ambient temperature (25 °C) for 30 min. Absorbance was recorded at 517 nm against a reagent blank using a microplate reader. The scavenging capacity was expressed as the concentration required to inhibit 50% of the DPPH radicals (IC_50_, µg/mL). All measurements were performed in triplicate.

#### 3.5.2. ABTS Radical Scavenging

The ABTS^•+^radical cation decolorization assay was carried out following the method of [93]. ABTS•^+^ was generated by reacting 2 mM ABTS stock solution with 2.45 mM potassium persulfate and allowing it to stand for 16 h at room temperature in the dark. The resulting solution was diluted with ethanol to achieve an absorbance of 0.700 ± 0.025 at 734 nm. For the assay, 40 µL of extract (having a concentration rage from 12.5 to 800 µg/mL) or standard (BHA and ascorbic acid) was added to 160 µL of the ABTS•^+^ working solution in each well of a 96-well plate. The mixture was incubated at room temperature for 10 min, after which the absorbance was measured at 734 nm. The IC_50_ values were determined from the percentage inhibition curves. Each sample was analyzed in triplicate.

#### 3.5.3. Reducing Power Assay (RP)

The reducing power of the extracts was assessed following the ferricyanide method [94], adapted to microplate format. Ten microliters of extract (3.125–200 µg/mL) or ascorbic acid standard were mixed with 40 µL of 0.2 M phosphate buffer (pH 6.6) and 50 µL of 1% potassium ferricyanide. After incubation at 50 °C for 20 min, 50 µL of 10% trichloroacetic acid was added. The mixture was combined with 40 µL distilled water and 10 µL of 0.1% ferric chloride. Absorbance was measured at 700 nm against a blank. Reducing power was expressed as A_0.5_, the extract concentration yielding an absorbance of 0.5, and compared with BHA and BHT. All tests were performed in triplicate.

#### 3.5.4. Superoxide Radical Scavenging (Alkaline DMSO Method)

Superoxide radical scavenging activity was determined using the alkaline DMSO method based on Kunchandy and Rao’s protocol [95]. In this assay, 30 µL of nitroblue tetrazolium (NBT, 1 mg/mL) or tannic acid standard, 40 µL of extract (12.5–800 µg/mL), and 130 µL of freshly prepared alkaline DMSO were mixed in a 96-well microplate. Ethanol was used as the blank, replacing the extract solution while following the same procedure. The reaction mixture was incubated at 25 °C for 5 min, and absorbance was measured at 560 nm against an appropriate blank. Each measurement was carried out in triplicate.

#### 3.5.5. Silver Nanoparticle (AgNP) Method

The antioxidant potential of the extracts was further evaluated using the silver nanoparticle (AgNP) formation method, following the previously described procedure of Mansur et al. [96]. Silver nanoparticles were prepared by reducing 1 mM silver nitrate with trisodium citrate. For the assay, 130 µL of freshly prepared AgNP solution, 50 µL of distilled water, and 20 µL of extract or ascorbic acid standard were mixed in a 96-well plate. After incubation in the dark at 25 °C for 30 min, absorbance was recorded at 423 nm. All measurements were performed in triplicate and the results were expressed as A0.5 values.

### 3.6. Enzyme Inhibitory Activities

#### 3.6.1. α-Amylase Inhibition

The α-amylase inhibitory activity was determined according to the method described by [97]. In a 96-well microplate, 25 µL of extract or acarbose standard at different concentrations (25–1600 µg/mL) was mixed with 50 µL of α-amylase solution (1 U) prepared in sodium phosphate buffer (pH 6.9, containing 6 mM NaCl). The mixtures were pre-incubated at 37 °C for 10 min. Subsequently, 50 µL of 1% soluble starch solution was added to initiate the reaction, followed by incubation for an additional 20 min at 37 °C. The reaction was terminated by adding 25 µL of 1 M HCl, and 100 µL of iodine–potassium iodide solution was added for color development. A control blank was prepared under the same conditions but without the enzyme. Absorbance was measured at 630 nm. The percentage inhibition of α-amylase activity was calculated using the following Equation (2):

%Inhibition = 1 − [(A_c_ − A_e_) − (A_s_ − A_b_)/(A_c_ − A_e_)] (2)

where A_c_ = absorbance of starch control (without enzyme), A_e_ = absorbance of enzyme reaction (without sample), A_s_ = absorbance of sample reaction, A_b_ = absorbance of sample blank (without enzyme).

#### 3.6.2. Urease Inhibition

Urease inhibitory activity was evaluated using the phenol–hypochlorite colorimetric method [98]. The reaction mixture contained 10 µL of extract (3.125–200 µg/mL), 25 µL of urease enzyme solution (5 U/mL), and 50 µL of 17 mM urea as substrate. After incubation at 30 °C for 50 min, phenol and hypochlorite reagents were added. Absorbance was measured at 630 nm. Thiourea was used as the reference inhibitor. All experiments were performed in triplicate and the results were expressed as IC_50_ values.

### 3.7. Sun Protection Factor (SPF) Estimation

The photoprotective capacity of the *R. officinalis* extracts was assessed using the in vitro method developed by Mansur et al. [99]. This method combines the principles of the erythemal action spectrum with spectrophotometric absorbance, covering the UV-B (290–320 nm) and UV-A II (320–340 nm) regions, which contribute significantly to skin erythema and sunburn [100].

Extract solutions were prepared at a concentration of 2 mg/mL in methanol, and their absorbance was measured at 5 nm intervals from 290 to 320 nm using a UV–Vis spectrophotometer with ethanol as the blank.

SPF values were calculated according to the following Equation (3):

(3) $$SPF=CF\times \sum_{290}^{320\;}\times \;EE\;(\lambda )\times I\;(\lambda )\times Abs\;(\lambda )$$

where CF is the correction factor (10), EE(λ) represents the erythemogenic effect of radiation at wavelength λ, I(λ) denotes the solar intensity at wavelength λ, Abs(λ) is the sample absorbance at each wavelength (Table 11).

### 3.8. Antimicrobial Activity

Antimicrobial activity was assessed using the disk diffusion method on Mueller–Hinton agar, following [102]. Paper disks (6 mm) impregnated with 35 µg of extract were placed on plates inoculated with test strains (*S. aureus*, *B. subtilis*, *E. coli*, *P. aeruginosa*, *C. albicans*). Zones of inhibition were measured after 48 h incubation at 37 °C.

### 3.9. Anti-Inflammatory Activity

#### 3.9.1. BSA Protein Denaturation Inhibition Assay (In Vitro)

The in vitro anti-inflammatory activity was determined by the bovine serum albumin (BSA) denaturation method as described by [100]. One milliliter of extract or reference drug (diclofenac sodium) was mixed with 1 mL of 0.2% BSA solution prepared in Tris-HCl buffer (pH 6.6). The mixture was incubated at 72 °C for 5 min, cooled to room temperature, and absorbance was measured at 660 nm. Tests were run in triplicate.

#### 3.9.2. Carrageenan-Induced Paw Edema (In Vivo)

The in vivo anti-inflammatory effect was evaluated using the carrageenan-induced paw edema model in Swiss albino mice (20–25 g) as described by [102]. Animals were divided into three groups (*n* = 5 per group). The test groups received an oral dose of the extract (10% *w*/*v*), and the reference group received diclofenac (10 mg/kg). After 30 min, 0.025 mL of 1% carrageenan solution was injected subplantarly into the left hind paw. After 4 h, the animals were euthanized, paws were dissected, and edema inhibition (%) was calculated relative to the untreated control group.

### 3.10. Evaluation of Analgesic Activity

The analgesic effect was investigated using the acetic acid-induced writhing test in mice, as described by [103], with reference to the procedure outlined by Meymandi (2013) [104]. Mice were randomly divided into control and treatment groups. Mice were randomly assigned to four groups: a negative control group, two treatment groups receiving the *R. officinalis* winter extract orally at doses of 100 and 500 mg/kg, and a positive control group receiving paracetamol orally at 500 mg/kg.

Each mouse received an intraperitoneal injection of acetic acid to induce abdominal constrictions (writhes). The number of writhes was recorded over a fixed observation period of 20–30 min. The percentage of analgesic protection was calculated using the following Equation (4):

(4) $$Protection\%=\frac{Mean\;writhes\;in\;control\;group\;-\;Mean\;writhes\;in\;treated\;group}{Mean\;writhes\;in\;control\;group}\times 100$$

A higher protection percentage was interpreted as stronger analgesic activity. All observations were conducted under identical conditions.

### 3.11. Acute Oral Toxicity Test

Acute oral toxicity was evaluated in compliance with OECD guideline 423 (Jonsson et al., 2013) [105]. Three groups of fasting mice (n = 5 per group) received a single oral dose of *R. officinalis* ethanolic winter extract at 275, 1562, and 2555 mg/kg, respectively. The animals were monitored daily for 14 days for signs of mortality, behavioral abnormalities, or any toxicological symptoms.

### 3.12. Cytotoxicity on Breast Cancer Cells (In Vitro)

The MCF-7 and MDA-MB-231 human breast cancer cell lines (ATCC, Penzberg, Germany) were cultured separately in 25 and 75 cm^3^ flasks using DMEM and RPMI-1640 mediums (Roswell Park Comprehensive Cancer Center, USA), supplemented with 10% fetal bovine serum (FBS), 1% L-glutamine, and 1% penicillin-streptomycin. Cultures were maintained at 37 °C in a humidified atmosphere with 5% CO_2_ and subcultured every 2–3 days upon reaching 80–90% confluency. Cells were detached using either 0.25% trypsin-EDTA solution or a sterile cell scraper, depending on the requirements of subsequent procedures.

Viability was determined using the Trypan blue dye exclusion method and counted manually under a light microscope. For cytotoxicity evaluation, 1.5 × 10^4^ cells per well were seeded into 96-well plates and allowed to adhere and stabilize for 24 h.

Extracts obtained from each seasonal sample were filtered, concentrated, and resuspended in DMSO. Working concentrations were then prepared in culture medium at 200, 100, 50, 25, 12.5, and 6.25 µg/mL.

After the 24 h stabilization period, cells were treated with the respective concentrations of seasonal *R. officinalis* extracts. Wells receiving only culture medium and solvent were designated as negative controls. Cytotoxicity was evaluated using the WST-1 assay (Roche Applied Science, Penzberg, Germany) at 48 and 72 h following treatment. Absorbance was measured at 450 nm with a reference wavelength of 630 nm using a microplate reader. The percentage of viable cells was calculated relative to the control group. All experiments were performed in triplicate, and data were analyzed to assess the time- and dose-dependent cytotoxic effects of *R. officinalis* extracts across different seasons on MCF-7 and MDA-MB-231 cell lines.

### 3.13. Statistical Analysis

Results are expressed as mean ± standard deviation (SD) from three independent analyses. Data were analyzed by one-way ANOVA followed by Tukey’s post hoc multiple comparison test. Principal component analysis (PCA) was used to evaluate the influence of season and the similarity between *R. officinalis* extracts in terms of biological activity and phenolic content. Pearson correlation analysis was then applied to determine relationships between these activities and phenolic compounds.

## 4. Conclusions

The findings obtained from this study confirm that seasonal variation is a fundamental environmental factor that clearly influences the chemical composition and biological activities of *R. officinalis* extracts. The analyses revealed that the accumulation of phenolic and flavonoid compounds in the plant parts does not occur uniformly throughout the year but rather follows cyclical fluctuations corresponding to the climatic and physiological changes associated with each season. These variations are attributed to the dynamic nature of biosynthetic processes, which are modulated by external factors such as temperature, solar radiation, and soil characteristics. These, in turn, affect the gene expression of enzymes responsible for the synthesis of biologically active secondary metabolites. The importance of seasonal influence lies not only in its impact on the total chemical composition but also on the biochemical quality of phenolic compounds, which directly determines the efficacy of the extracts in counteracting oxidative and inflammatory stress, as well as in inhibiting key metabolic enzymes. It was observed that certain seasons yielded extracts with enhanced antioxidant activity, while others were more effective in anti-inflammatory or enzyme inhibitory activities. This indicates that the distribution of bioactive compounds is specialized and season-dependent, and cannot be accurately predicted through simple quantitative analysis alone. These results are of significant importance not only from a botanical and biochemical perspective but also in terms of practical applications in the pharmaceutical, cosmetic, and food industries. Optimizing the harvest time emerges as a strategic step to ensure the extraction of plant materials with high biological efficacy, thereby enhancing the quality and stability of natural products derived from medicinal plants. Furthermore, the findings underscore the need to integrate seasonal analysis into plant valorization studies and to reconsider certain traditional practices that overlook the temporal dimension of plant collection and use. Based on these insights, the study recommends adopting an integrated approach for evaluating medicinal plants, one that incorporates environmental and seasonal factors alongside conventional chemical and biological analyses. It also encourages further research into the qualitative characterization of phenolic compounds across different seasons and the investigation of their molecular mechanisms of action in biological models. Such an approach could lead to the development of innovative and sustainable applications based on local plant resources such as *R. officinalis*.

## Figures and Tables

**Table 1:** Seasonal LC-ESI-MS/MS analysis of *R. officinalis* extracts.

No	Compounds	RT (min)	Autumn Extract (RAutumn) (µg/g)	Winter Extract (RWinter) (µg/g)	Spring Extract (RSpring) (µg/g)	Summer Extract (Rsummer) (µg/g)
1	gallic acid	2.468	4.80	3.53	1.48	2.801
2	epigallocatechin	2.520	13.50	ND	ND	ND
3	chlorogenic acid	2.531	2.27	1.63	5.49	1.44
4	catechin	2.729	3.89	ND	ND	ND
5	gentisic acid	2.711	8.78	14.40	ND	ND
6	caffeic acid	3.216	2.77	15.49	7.63	3.97
7	syringic acid	3.310	2.04	3.20	6.41	2.52
8	vanillic acid	3.781	ND	ND	ND	ND
9	rutin	2.950	64.05	ND	4.79	11.65
10	isoquercitrin	4.796	6.77	2.21	8.67	1.00
11	polydatin	4.438	ND	ND	ND	ND
12	4-hydroxybenzaldehyde	4.551	0.13	ND	ND	ND
13	*p*-coumaric acid	4.453	ND	5.10	ND	ND
14	sinapic acid	5.097	ND	ND	ND	ND
15	vanillin	5.197	5.60	ND	10.32	4.32
16	*trans*-ferulic acid	4.928	6.55	16.63	12.11	5.57
17	taxifolin	5.110	ND	ND	ND	ND
18	salicylic acid	7.214	10.78	3.06	3.87	ND
19	*o*-coumaric acid	8.216	ND	ND	ND	ND
20	baicalin	7.949	ND	ND	ND	ND
21	protocatehuic ethyl ester	8.484	ND	ND	ND	ND
22	protocatechuic acid	8.497	ND	2.07	1.44	ND
23	kaempferol	9.798	7.67	21.65	33.75	15.92
24	resveratrol	10.511	ND	ND	ND	ND
*25*	*trans*-cinnamic acid	11.173	ND	7.68	9.33	ND
26	naringenin	11.447	ND	ND	ND	ND
27	morin	11.840	2.10	14.70	7.62	3.06
28	quercetin	11.840	2.64	16.82	8.72	3.80
29	7-hydroxyflavone	11.969	ND	ND	ND	ND
30	chrysin	13.578	ND	ND	ND	ND
31	luteolin	13.633	86.90	38.28	15.37	52.32
32	biochanin a	13.752	86.80	82.86	91.37	84.86
33	5-hydroxyflavone	15.272	1.07	2.48	2.90	2.03
34	diosgenin	20.281	ND	ND	ND	ND

Rt: Retention time; ND: Not detected.

**Table 2:** IC_50_ (µg/mL) and A_0.5_ (µg/mL) values of antioxidant activity for the four seasonal *R. officinalis* extracts.

	IC_50_ (µg/mL)			A_0.5_ (µg/mL)	
	DPPH	ABTS	ADS	FRAP	SNP
Spring extract (RSpring)	41.09 ± 0.15 ^c^	48.27 ± 2.74 ^e^	42.28 ± 0.03 ^b^	7.62 ± 0.30 ^a^	35.56 ± 1.74 ^d^
Winter extract (RWinter)	114.42 ± 4.38 ^d^	37.41 ± 0.97 ^c^	42.95 ± 0.07 ^b^	48.74 ± 2.54 ^c^	71.95 ± 0.31 ^e^
Autumn extract (RAutumn)	24.72 ± 0.16 ^b^	14.76 ± 0.60 ^b^	41.89 ± 0.01 ^b^	26.36 ± 1.44 ^b^	17.96 ± 0.44 ^b^
Summer extract (Rsummer)	41.51 ± 2.13 ^c^	42.49 ± 0.94 ^d^	44.63 ± 0.23 ^c^	25.37 ± 5.56 ^b^	27.46 ± 0.33 ^c^
BHA	6.89 ± 0.12 ^a^	1.91 ± 0.09 ^a^	NT	NT	NT
Acid ascorbic	22.11 ± 0.78	14.15 ± 1.1	NT	6.77 ± 1.15 ^a^	7.14 ± 0.05 ^a^
Tannic acid	NT	NT	3.125± 0.003 ^a^	NT	NT

Standard, NT: not determined. Means within a column sharing the same superscript letter are not significantly different, while those with different superscript letters differ significantly (*p* < 0.05), according to Tukey’s HSD test.

**Table 3:** IC_50_ values (µg/mL) for α-amylase inhibition by seasonal *R. officinalis* extracts.

Specification		Alpha-Amylase (IC_50_ µg/mL)
Ethanolic extract	Spring extract (RSpring)	1171.73 ± 19.25
	Winter extract (RWinter)	>1600
	Autumn extract (RAutumn)	1142.04 ± 11.32
	Summer extract (Rsummer)	>1600
Standard	Acarbose	3650.93 ± 10.70

**Table 4:** IC_50_ values (µg/mL) for urease inhibition by seasonal *R. officinalis* extracts.

Specification		Urease (IC_50_ µg/mL)
Ethanolic extract	Spring extract (RSpring)	62.26 ± 0.58
	Winter extract (RWinter)	NA
	Autumn extract (RAutumn)	NA
	Summer extract (Rsummer)	NA
Standard	Thiourea	11.57 ± 0.68

NA: no activity.

**Table 5:** SPF values of seasonal *R. officinalis* extracts.

Extract	Spring Extract (RSpring)	Winter Extract (RWinter)	Autumn Extract (RAutumn)	Summer Extract (Rsummer)
SPF	30.79 ± 0.52 ^a^	25.18 ± 0.32 ^a^	30.97 ± 0.42 ^a^	32.46 ± 0.35 ^a^

^a^ homogeneous groups (*p* ˂ 0.05) as determined by Tukey’s HSD post hoc test.

**Table 6:** Inhibition zone diameters (mm) of bacterial and yeast strains at various concentrations of seasonal *R. officinalis* extracts.

Strains Used	Seasons	80 mg/mL	40 mg/mL	20 mg/mL	10 mg/mL	GNT
*Escherichia coli* ATCC 25922	autumn	NI	NI	NI	NI	27
	winter	NI	NI	NI	NI	27
	spring	NI	NI	NI	NI	26
	summer	NI	NI	NI	NI	26
*Pseudomonas aeruginosa* ATCC 27853	autumn	NI	NI	NI	NI	26
	winter	NI	NI	NI	NI	26
	spring	NI	NI	NI	NI	26
	summer	NI	NI	NI	NI	26
*Staphylococcus aureus* ATCC 25932	autumn	13	10	9	8	31
	winter	9	NI	NI	NI	32
	spring	9	8	8	NI	33
	summer	9	8	8	7	31
*Bacillus subtilis* ATCC 25973	autumn	7	NI	NI	NI	24
	winter	NI	NI	NI	NI	24
	spring	NI	NI	NI	NI	24
	summer	NI	NI	NI	NI	23
*Candida albicans* ATCC 10231	autumn	NI	NI	NI	NI	/
	winter	NI	NI	NI	NI	/
	spring	NI	NI	NI	NI	/
	summer	NI	NI	NI	NI	/

NI = No Inhibition, GNT = Gentamicin.

**Table 7:** IC_50_ values (µg/mL) of the in vitro anti-inflammatory activity of *R. officinalis* extracts according to season.

Specification		IC_50_ (µg/mL)
Ethanolic extract	Spring extract (RSpring)	106.01 ± 6.17 ^c^
	Winter extract (RWinter)	28.60 ± 2.84 ^a^
	Autumn extract (RAutumn)	107.78 ± 2.09 ^d^
	Summer extract (Rsummer)	125.61 ± 1.22 ^b^
Standards	Diclofenac^®^	40.90 ± 0.89 ^b^

^a–d^ homogeneous groups (*p* ˂ 0.05) as determined by Tukey’s HSD post hoc test.

**Table 8:** Anti-inflammatory activity results of the winter extract of *R. officinalis.*

Treatment	Dose (mg/kg)	Percentage of Edema %	Percentage of Inhibition %
Control	---	51.80 ± 1.92 ^d^	---
Diclofenac	500	14.00 ± 1.58 ^a^	72.97
*R. officinalis*	100	22.80 ± 1.48 ^c^	55.98
	500	19.20 ± 1.48 ^b^	62.93

Means within a column sharing the same superscript letter are not significantly different, while ^a–d^ homogeneous groups (*p* ˂ 0.05) as determined by Tukey’s HSD

**Table 9:** Analgesic activity results of the winter extract.

Treatment	Dose (mg/kg)	Number of Cramps	Percentage of Protection %
Control	---	69.00 ± 3.16 ^c^	---
Paracetamol	500	20.40 ± 1.94 ^a^	70.43
*R. officinalis*	100	29.20 ± 1.30 ^b^	57.68
	500	22.00 ^d^ ± 1.68	68.41%

^a–d^ homogeneous groups (*p* ˂ 0.05) as determined by Tukey’s HSD post hoc test.

**Table 10:** IC_50_ values (µg/mL) of *R. officinalis* extracts on MCF-7 (a) and MDA-MB-231 cell lines.

Extracts	MCF-7 (IC_50_, µg/mL)	MDA-MDB-231 (IC_50_, µg/mL)
Spring extract (RSpring)	147.68	84.85
Winter extract (RWinter)	119.71	122.46
Autumn extract (RAutumn)	178.98	180.95
Summer extract (Rsummer)	115.94	114.02

**Table 11:** Standard erythemogenic effect and solar intensity factors (EE(λ) × I(λ)) at selected UV wavelengths used for SPF calculation according to [101].

Wavelength (nm)	EE(λ) × I(λ)
290	0.0150
295	0.0817
300	0.2874
305	0.3278
310	0.1864
315	0.0839
320	0.0180

## Data Availability

The original contributions presented in the study are included in the article; further inquiries can be directed to the corresponding author.
